# Volumetric, Compressibility and Viscometric Approach to Study the Interactional Behaviour of Sodium Cholate and Sodium Deoxycholate in Aqueous Glycyl Glycine

**DOI:** 10.3390/molecules27248998

**Published:** 2022-12-16

**Authors:** Santosh Kumari, Suvarcha Chauhan, Kuldeep Singh, Ahmad Umar, Hassan Fouad, Mohammed S. Alissawi, Mohammad Shaheer Akhtar

**Affiliations:** 1 Department of Chemistry, Himachal Pradesh University, Summer Hill, Shimla 171005, India; 2 Department of Chemistry, MCM DAV College, Kangra 176001, India; 3 Department of Chemistry, College of Science and Arts, and Promising Centre for Sensors and Electronic Devices (PCSED), Najran University, Najran 11001, Saudi Arabia; 4 Department of Materials Science and Engineering, The Ohio State University, Columbus, OH 43210, USA; 5 Applied Medical Science Department, Community College, King Saud University, Riyadh 11433, Saudi Arabia; 6 College of Engineering, King Saud University, Riyadh 11433, Saudi Arabia; 7 School of Semiconductor and Chemical Engineering, Jeonbuk National University, Jeonju 54896, Republic of Korea; 8 Graduate School of Integrated Energy-AI, Jeonbuk National University, Jeonju 54896, Republic of Korea

**Keywords:** intermolecular interactions, glycyl glycine, bio-surfactants, apparent molar volume, isentropic compressibility, apparent molar adiabatic compression

## Abstract

Viscosity, speed of sound (*u*), and density (*ρ*) have been measured in aqueous glycyl glycine solution over a temperature range from 293.15 to 313.15 K with a 5 K interlude to evaluate the volumetric and compressibility properties of bio-surfactants, namely sodium cholate (NaC; 1–20 mmol∙kg^−1^) and sodium deoxycholate (NaDC; 1–10 mmol∙kg^−1^). Density and viscosity findings provide information on both solute–solute and solute–solvent types of interactions. Many other metrics, such as apparent molar adiabatic compression $\left(\kappa_{S,\varphi }\right)$, isentropic compressibility $\left(\kappa_{S}\right)$, and apparent molar volume $\left(V_{\varphi }\right)$, have been calculated from speed of sound and density measurements, utilising experimental data. The results show that the zwitterionic end group in the glycyl glycine strongly interacts with NaDC and NaC, promoting its micellization. Since the addition of glycyl glycine causes the bio-surfactant molecules to lose their hydrophobic hydration, the observed concentration-dependent changes in apparent molar volume and apparent molar adiabatic compression are likely attributable to changes in water–water interactions. Viscous relaxation time (τ) increases significantly with a rise in bio-surfactant concentration and decreases with increasing temperature, which may be because of structural relaxation processes resulting from molecular rearrangement. All of the estimated parameters have been analysed for their trends with regard to the different patterns of intermolecular interaction present in an aqueous glycyl glycine solution and bio-surfactant system.

## 1. Introduction

Surfactants are a class of compounds that tend to form aggregates of different forms owing to the presence of polar and non-polar groups present in the same molecules. These aggregates are formed in a solution at a certain concentration, called the critical micelle concentration, i.e., CMC. This is a significant property associated with surfactants which is caused by the hydrophobic interactions of the hydrocarbon tail with water, balanced by electrostatic repulsions between the surfactants head groups [1,2]. Bio-surfactants comprise a wide array of amphiphilic molecules and offer many advantages, for instance high biodegradability and low toxicity, as well as remaining active at high pH and salinity [3]. Bio-surfactants exhibit complex behaviours compared to conventional surfactants. Sodium cholate (NaC) and sodium deoxycholate (NaDC) are examples of bio-surfactants that are present naturally in the bile of mammals and other vertebrates; they are anionic, stiff, and steroidal and play an important role in a wide variety of biological activities [4,5,6]. In the digestive tract, they serve as an emulsifier and solubilizer and are essential components of steroidal detergents. Bio-surfactants/bile salts are produced in the liver from cholesterol and are kept in the gallbladder. The steroid framework of bile salt molecules is amphipathic, with both convex and concave faces visible [7,8]. Hydrogen and methyl groups on the convex side make it hydrophobic; hydroxyl groups on the concave side make it hydrophilic. These competing functions are evident in their dissimilar aggregation and solubilization behaviour, and are hypothesised to be a result of subtle architectural differences in their chemical structure, particularly in their bile acid moiety. Thus, the micellization of two bio-surfactants, NaC and NaDC, that vary by a single –OH group on the bile acid moiety, was evaluated using a number of methods and by observing their aggregation behaviour in the presence of several additives [9].

On the other hand, proteins, being most versatile and complex molecules, are characteristically longer chains of amino acids linked by peptide bonds and their functioning is entirely subject to their structure [10,11,12]. They are essential for all cellular metabolic processes. Proteins play a crucial role in cellular chemistry and are required for the majority of cellular processes in all living organisms, contributing to structural integrity and the carrying of materials, among other things. In contrast, enzymes, hormones, and antibodies are all examples of peptides, which are short polymers connected by typically less than or equal to 100 amino acids. They are crucial for the production of proteins and play a vital role in this process [13]. Peptide interactions with the many metabolites in a living organism are diverse and important to their physiological effect. For a comprehensive understanding of the stability and association of bio-molecules including peptides, sugars, amino acids, and proteins in aqueous environments, as well as the capacity to discern between interactions, a systematic understanding of the solution behaviour of these compounds is required [14,15,16,17,18,19]. Therefore, it is useful for elucidating the process of protein self-aggregation, which continues to be the driving force behind a wide variety of biological interactions.

Electrostatic and hydrophobic interactions have recently been hypothesised as the primary mechanisms for the connection of bio-active and bio-surfactants molecules in aqueous solutions, including proteins, peptides, and amino acids. Solvent type also plays an important role and may influence micellar system stability by either partitioning the micellar and aqueous phase or accumulating within the micellar core [20,21,22,23]. However, electrolyte disturbs the structure of the solvent which also has a direct impact on the micellization.

In this context, we evaluate the viscosity, speed of sound and density of bio-surfactants such as NaDC and NaC at various temperatures to acquire a sense of how aqueous glycyl glycine affects their micellization. Apparent molar volume, apparent molar isentropic compression, and isentropic compressibility have been computed using experimental speed of sound and density data, and various electrostatic and hydrophobic interactions relevant to the ternary (bio surfactant–glycylglycine–water) system were used to explain the findings. Using experimental viscosity data, the viscous relaxation time and relative viscosity have been estimated. Their dependency on glycylglycine content and temperature indicated the presence of peptide–surfactant interactions. It is a well-known fact that sodium or potassium salts of cholic and deoxycholic acids (bile salts) are useful solubilisation agents for various large molecules, including enzymes (proteinaseous). Therefore, the present study may be very helpful to analyse the conformational alterations that may occur through bile salt–protein interactions.

## 2. Results and Discussion

### 2.1. Volumetric and Compressibility Studies

**Figure 1:**
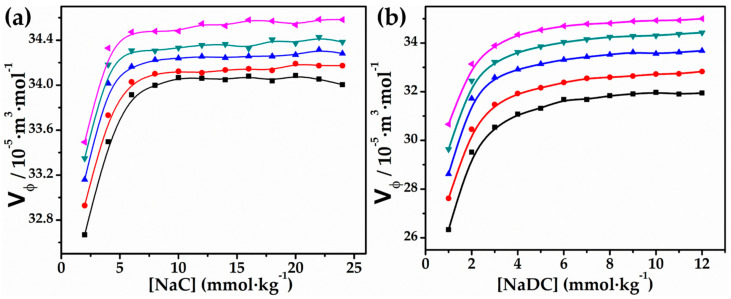
Plots of $V_{\varphi }$ vs. (**a**) [NaC] and (**b**) [NaDC] in 0.010 mol∙kg^−1^ aqueous solution of glycyl glycine at 293.15 K (■), 298.15 K (●), 303.15 K (▲), 308.15 K (▼), and 313.15 K (◄).

The molecules’ physicochemical behaviour as well as structural rearrangement in a solution system may be understood by basic techniques, such as measuring density along with the speed of sound. This work measures the speed of sound and density in aqueous solutions at 293.15–313.15 K temperature, with intervals of 5 K to investigate the impact of glycyl glycine on the volumetric and compressibility characteristics of the bio-surfactants NaDC and NaC. Tables S1 and S2 in the Supplementary Materials provides the density as well as speed of sound data, respectively. Various metrics, such as apparent molar adiabatic compression $\left(\kappa_{S,\varphi }\right)$, isentropic compressibility $\left(\kappa_{S}\right)$, and apparent molar volume have been gained from the experimental data on speed of sound along with density. Without a doubt, the composition of the solvent surrounding the solute species determines all the aforementioned characteristics, and these parameters carry information about the structural repercussions of solute–solvent interactions. Both NaDC and NaC $V_{\phi }$ values were determined utilizing Equation (1) [24,25,26,27,28]:

(1) $$V_{\phi }=\frac{M}{\rho }+\frac{\left[\rho_{o}-\rho \right]}{m\rho \rho_{o}}$$

where *M* (kg·mol^−1^) defines the molar mass of the solute (NaC/NaDC), and $\rho_{o}$ and ρ are the densities of the solvent and solution, respectively. Table 1 shows the computed values for NaDC and NaC in both an aqueous solution of glycyl glycine and pure water. Figure 1 shows how the apparent molar volume changes as a function of NaDC and NaC concentrations for 0.010 mol∙kg^−1^ glycyl glycine over a wide range of temperatures. Because of their non-linear effect on the concentrations of both bio-surfactants [29], the data for the whole concentration range examined could not be analysed using Masson’s equation $V_{\phi }=V_{\phi }^{o}+S_{v}^{*}m^{1/2}$. $V_{\phi }$ values may vary by as much as ±0.2 × 10^−5^ m^3^∙mol^−1^ due to experimental errors. All of the characteristics are influenced by the way solvent molecules interact with solute molecules, thus they are recognized to include data on the structural repercussions of the solution mixture that cause the interactions between the solute and solvent [30,31,32,33,34]. The examined system is hypothesised to include a number of different types of relations between the appropriate groups of NaDC and NaC and glycyl glycine monomers. The following drawings may be used to depict these interactions:

(a) Hydrophobic–hydrophobic interactions between the hydrophobic part of NaC/NaDC and the non-polar part of the glycylglycine,
(b) Hydrophobic–hydrophilic interactions between the hydrophobic part NaC/NaDC and the hydrophilic groups of glycyl glycineor viceversa,
(c) Hydrogen bonding and other hydrophilic–hydrophilic interactions link the hydrophilic groups of NaC/NaDC to the hydrophilic groups of gylycyl glycine,
(d) Ion–ion interactions between the glycylglycine –COO^−^/NH^3+^ ions and the polar region of NaC/NaDC.

Apparent molar volume $\left(V_{\phi }\right)$ magnitudes are largely determined by the nature of these interactions, which alter the solvent’s structural arrangement. The co-sphere overlap method proposed by Friedman and Krishnan [35] suggests that interactions are a deciding factor in determining the magnitude of the values of apparent molar volumes $V_{\phi }$, but even a rise in the electrostriction, which may be due to the first two kinds of interactions, will lead to the disruption of the water structure and, thus, a decrease in the $V_{\phi }$ values. Alternatively, the interactions involved in reducing the electrostatic contacts—namely, ion–ion and hydrophilic–hydrophilic interactions—lead to improvements in water molecule structure. Therefore, higher $V_{\phi }$ values are seen. These interactions are also thought to be responsible for the positive volume contribution made by bio-surfactants, since they reduce the electrostriction of water molecules near their ionic head groups. Increased $V_{\phi }$ values are also seen for bio-surfactants, because such interactions may lead to the degradation of structured water molecules around the steroidal backbone of bio-surfactants, which would increase the volume of solution [36,37].

Positive results were reported for all temperatures and concentrations (Table 1) across all experimental circumstances, indicating the occurrence of type 3 as well as type 4 interactions between the various species. At low concentrations, the $V_{\phi }$ values for both NaDC and NaC increase suddenly with reasonable magnitude, changing to slightly curved or linear as the surfactant concentration is raised beyond a certain concentration, (above CMC), for all solvent systems studied at various temperatures. As with NaC/NaDC, the positive $V_{\phi }$ values for both bile salts grow at lower NaC/NaDC concentrations (≈14 mmol∙kg^−1^ for NaC and 5 mmol∙kg^−1^ for NaDC, respectively), but they plateau at higher concentrations (i.e., after the CMC was met). The existence of the aforementioned particular interactions, in addition to certain non-specific interactions, may shed light on this kind of behaviour at low concentrations [38]. Hydrophobic interactions prevail at higher concentrations, whereas particular interactions, ideally electrostatic in nature, and non-specific interactions play a role in the lower concentration range [39]. This kind of behaviour has been shown by both our previous bile salt experiments [40,41] and those of other researchers for the typical surfactant [42,43]. The binding of the counter ion to the micelle not only produces structural changes because of the electrostatic repulsion among NaC/NaDC and bio-surfactant’s head groups, but also due to the release of structured water around the hydrophobic region or from the counter ion [44,45]. Due to a rise in strong solute–solvent interactions upon addition of glycyl glycine solution, the magnitude of the value $V_{\phi }$ rises with temperature and [glycyl glycine] in the following order: pure water 0.010 > 0.005 > 0.001 mol∙kg^−1^ aqueous solution of glycyl glycine [46]. The $V_{\phi }$ values for NaC have been found to be of greater magnitude in aqueous solution glycyl glycine compared to NaDC. This observation can be explained in terms of the more hydrophobic nature of NaC, which causes easier micellization that is accompanied by expulsion of water molecules from the aggregated structure (micelle) of surfactant, leading to larger $V_{\phi }$ values.

The compressibility of the solution for the various systems under study has been discussed using measured density and speed of sound values. Following equation [47] yields a list of values for the compressibility parameter, apparent molar isentropic compression $\left(\kappa_{s,\phi }\right)$.

(2) $$\kappa_{S,\phi }{}_{}=V_{\phi }\kappa_{s}+\frac{\left[\kappa_{s}-\kappa_{o}\right]}{m\rho_{o}}$$

where the solution’s molality is defined by *m*, which is determined through the equation [48] from the molar concentration values.

*m* =1/[*ρ*/*C* − *M*/1000] (3)

where molar concentration is defined by *C*, the surfactant’s relative molar mass is defined by *M*, *ρ_o_* and *ρ* are the densities of pure solvent and solution, respectively, the solvent’s isentropic compressibility is defined by $\kappa_{o}$, and the solution’s isentropic compressibility is defined by $\kappa_{s}$. Values of $\kappa_{s}$ and $\kappa_{o}$ were measured as [49]:

(4) $$\kappa_{s}=1/u^{2}\rho$$

and

(5) $$\kappa_{o}=1/{u_{o}}^{2}\rho_{o}$$

**Figure 2:**
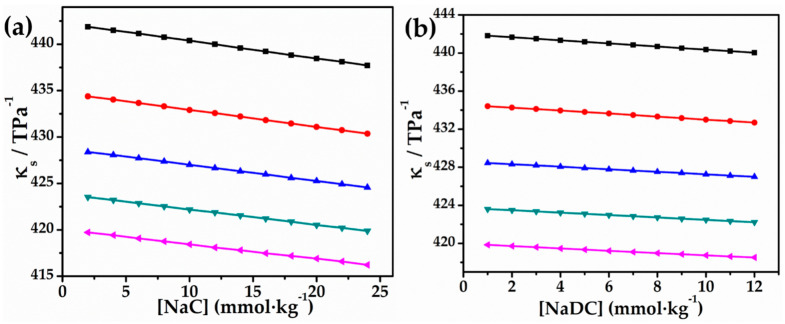
Plots of $\kappa_{s}$ vs. (**a**) [NaC] and (**b**) [NaDC] in 0.010 mol∙kg^−1^ aqueous solution of glycyl glycine at 293.15 K (■), 298.15 K (●), 303.15 K (▲), 308.15 K (▼), and 313.15 K (◄).

Isentropic compressibility $\kappa_{s}$ values for NaDC and NaC in glycyl glycine aqueous solutions are shown in Table 2. Figure 2 illustrates the typical distribution curves for NaDC and NaC in 0.010 mol∙kg^−1^ glycyl glycine solutions in water. The primary result from compressibility is the extent to which the $\kappa_{s}$ concentration of both bio-surfactants investigated drops with increasing temperature. As temperature rises, the $\kappa_{s}$ values drop, perhaps because the water structure surrounding the glycyl glycine zwitterions and the hydrophilic groups of NaDC and NaC are broken, increasing the NaC/NaDC–glycyl glycine interactions [50]. Table 2 explores further to reveal that the $\kappa_{s}$ values of both bio-surfactants are dependent on the hydrophobic group size and glycyl glycine concentration. Prominent information gained from Figure 2 is that $\kappa_{s}$ decreases quite significantly with the concentration of both the bio-surfactants, which shows that they behave differently from common electrolytes [51,52,53]. This observation can be understood in terms of the effect of additives in solution. On addition of bile salt, the incompressibility of the solution increases because of the higher number of incompressible species. Due to the presence of strong solute–solvent interaction, it becomes hard to compress the solution, causing a decrease in the isentropic compressibility value. Moreover, the solute–solvent interactions may lead to the aggregation of bile salt molecules to form micelles and, consequently, the solution becomes hard to compress which also leads to lower $\kappa_{s}$ values. In addition to this, temperature is also going to influence $\kappa_{s}$ values. The $\kappa_{s}$ values decrease with temperature, which may be ascribed to the fact that increased temperature breaks the structured water cage around the solute molecules and enhances the feasibility of the dipeptide’s interaction with the bile salt that ultimately results in lower $\kappa_{s}$ values. We discovered that the $\kappa_{s}$ levels of bio-surfactants in glycyl glycine aqueous solutions decreased in order as NaC > NaDC. It is important to note that the hydrophobic groups in this decreasing sequence grew progressively larger, leading to stronger interactions between bio-surfactants and glycyl glycine as the sequence decreased.

**Figure 3:**
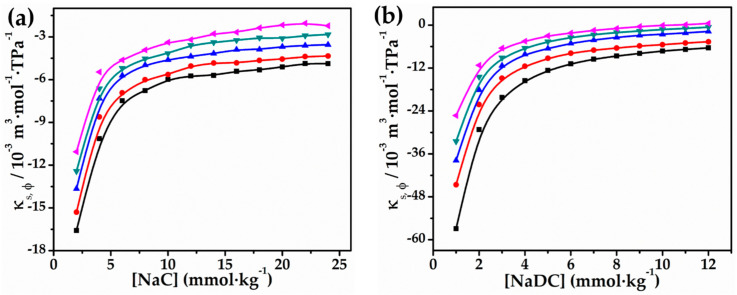
Plots of $\kappa_{s,\varphi }$ vs. (**a**) [NaC] and (**b**) [NaDC] in 0.010 mol∙kg^−1^ aqueous solution of glycyl glycine at 293.15 K (■), 298.15 K (●), 303.15 K (▲), 308.15 K (▼), and 313.15 K (◄).

One more crucial characteristic is that $\kappa_{s,\phi }$ may provide decisive evidence concerning the interactions occurring in the NaC/NaDC–glycyl glycine system. The $\kappa_{s,\phi }$ values have been presented in Table 3 and the variation of $\kappa_{s,\phi }$ values as a function of [NaC/NaDC] for 0.010 mol∙kg^−1^ glycyl glycine have been provided in Figure 3. The findings show a similar variation to the apparent molar volume and are consistent with the variation of $\kappa_{s,\phi }$ with the concentration of bio-surfactants for all of glycyl glycine’s concentrations and temperatures. Moreover, the results are negative for the solvent systems examined, and their magnitude diminishes with an increasing concentration of NaC/NaDC and glycylglycine. Various phenomena, such as hydrophobic solvation and electrostriction, are brought about by the negative values for apparent molar isentropic compression $\kappa_{s,\phi }$, which shows that solvent molecules are compressed less tightly around the bio-surfactant molecules than they are in the bulk solution.

The inability of the surrounding solvent molecules to be compressed as a result of the presence of significant electrostrictive forces is what causes electrostrictive solvation to occur. A rise in the concentration of glycyl glycine was shown to be associated with the observation of reduced negative values, which are also referred to as increase values. This may be because a rise in the glycyl glycine molecule concentration in the hydrophobic micellar region is connected with a rise in the number of voids or free space, both of which lead to higher $\kappa_{s,\phi }$ values [54]. In addition, the presence of a glycyl glycine molecules are responsible for disruption of the organised water on the NaC/NaDC surface, resulting in strengthened water–glycyl glycine interactions by hydrogen bonding [55]. These might all explain why bio-surfactants in a water–glycyl glycine solvent solution have smaller absolute values of $\kappa_{s,\phi }$ compared to those in pure water [56]. Figure 3 also shows that the rise in $\kappa_{s,\phi }$ values is steep up to a specific concentration (micellar area), but becomes practically linear after CMC, which is consistent with the observation that the fluctuation in apparent molar volume flattens out after CMC. This proves that hydrophobic interactions predominate at greater concentrations for both bio-surfactants, whereas hydrophilic interactions predominate at lower concentrations. The significance of hydrophobic interactions in micelle formation is further supported by this $\kappa_{s,\phi }$ kind of behaviour. These results are congruent with those that were discovered in earlier research that was published [57].

### 2.2. Viscometric Studies

In this section, the influence of bio-surfactants (NaC/NaDC) on the structure of aqueous glycyl glycine solutions was investigated by measuring their viscosity at 293.15–318.15 K temperature in solutions containing 0.001, 0.005, and 0.010 mol∙kg^−1^ glycyl glycine. Viscosity, the inner resistance in the fluid/liquid, is the most noteworthy of transport properties, and is affected by several factors viz., temperature, the shape of the molecules and the molecular weight. The viscosity depends upon the strength of intermolecular interactions and the arrangement of the molecules present in the system [58,59,60,61]. Moreover, to understand these interactions in various industrial and engineering operations for the validation of pharmacological and biotechnological processes, the study of viscosity measurements has become indispensable. Table S3 of the Supplementary Materials presents the viscosity information of the bio-surfactants in glycyl glycine aqueous solutions. Viscosity values have been calculated by using the given equation:

(6) $$\eta =\eta_{o}\frac{\rho \times t}{\rho_{o}\times t_{o}}$$

where, $\eta_{o}$ is defined as solvent’s viscosity, $t_{0}$ is the flow time of the solvent and $\rho_{o}$ is the solvent’s density, while $\eta_{}$ is the system’s viscosity, t is the system’s flow time, and ρ is defined as the system’s density.

On an inspection of viscosity data, it was determined that the η values rise with an increase in the bio-surfactants’ concentration along with the glycyl glycine’ concentrations, which may be ascribed to intermolecular interactions existing in the solution that are electrostatic as well as hydrophobic in nature. Fascinatingly, viscosity values show significant variation within the specific concentration range (~CMC) for both the bio-surfactants. This type of behaviour gives confirmation of structural switches, which may be due to the micellization process of these bio-surfactants in the aqueous glycyl glycine solutions. However, as the temperature increases, the intermolecular connections decrease because of the increased kinetic energy of the molecules, and the viscous force weakens as a result [62,63,64]. For the bio-surfactants, the order remains as NaDC > NaC, which is as expected because of the greater hydrophobic character of NaDC, and thereby facilitates micellization/aggregation to a greater extent [65].

In addition, relative viscosity, $\eta_{r}$, was calculated utilizing viscosity measurements by putting the numbers into the equation [66]

(7) $$\eta_{r}=\frac{\eta }{\eta_{0}}$$

**Figure 4:**
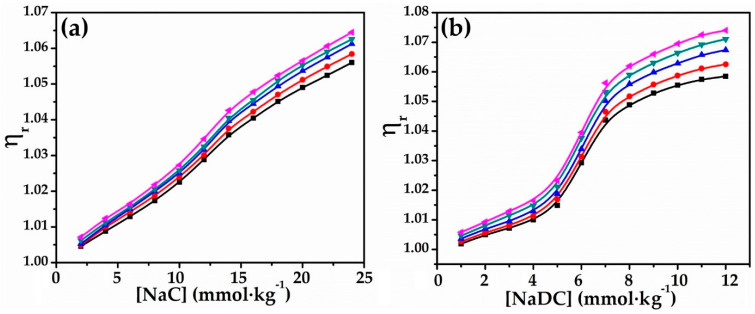
Plots of $\eta_{r}$ vs.(**a**) [NaC] (**b**) [NaDC] in 0.010 mol∙kg^−1^ aqueous solution of glycyl glcine at 293.15 K (■), 298.15 K (●), 303.15 K (▲), 308.15 K (▼), and 313.15 K (◄).

Relative viscosity results at various glycyl glycine concentrations in water are listed in Table 4, and graphs of relative viscosity vs. bio-surfactants are displayed in Figure 4. It has been noted from the plots that relative viscosity shows a steady increase at lower concentrations of bio-surfactants (<14 mmol∙kg^−1^ for NaC and <5 mmol∙kg^−1^ for NaDC), but escalates abruptly at higher concentration [67,68]. Interestingly, this observation of the results of the relative viscosity is consistent with the above discussed variations in viscosity measurements.

Viscous relaxation time τ for bio-surfactants in aqueous solutions of glycyl glycine at various temperatures were indexed in Table 5 and estimated using the equation given below [69]:

(8) $$\tau =\frac{4}{3}\frac{\eta }{u^{2}\rho }$$

where η is defined as the viscosity, ρ is defined as the density, and u is defined as solution’ speed sound. To learn more about the nature of the system’s intermolecular interactions, scientists have employed the viscous relaxation time, which can be derived from the system’s viscosity using the above equation. The time required for the excitation energy to be converted into translational energy depends on the ambient temperature, as well as any impurities [70]. This direct transfer of excitation energy into translational energy is more proof that structural relaxation processes occur, and it is therefore temperature dependent. It has been recorded that τ values vary with the concentration of the surfactant in a similar fashion to the relative viscosity $\eta_{r}$ of the studied system. From the data on viscous relaxation time, it has been clearly seen that τ values rise gradually when increasing the concentrations of both bio-surfactants used, as well as with the concentration of glycyl glycine, and fall off with temperature. Structural relaxation mechanisms are important contributors to the rearrangement of molecules in the system under investigation. Moreover, the τ values for bio-surfactants have been found to be of higher magnitude for NaDC (within experimental error) than that of NaC in aqueous solutions of glycyl glycine. These results strengthen the results obtained from earlier studies on micellization and clearly reflect the structural relationship, i.e., the more hydrophobic nature of the surfactants.

## 3. Experimental Details

### 3.1. Materials

All of the tests were performed using deionized water that was distilled in a Millipore–Elix system (Burlington, MA, USA) and had (2 to 3) × 10^−6^ S∙cm^−1^ conductivity and 6.8–7.0 pH at a temperature of 298.15 K. Himedia Pvt. Ltd. (Mumbai, India) supplied us with NaC and NaDC of AR quality. Ltd. (Mumbai, India), and then recrystallized from ethanol using the strategy described in our prior publications [10,71]. The glycylglycine has been acquired from Spectrochem Pvt. Ltd. (Mumbai, India), and has been put to regular usage without any special handling. The details of the used chemicals in this study are presented in Table 6.

### 3.2. Methods

An Anton Paar DSA-5000 device (Graz, Austria) has been utilized for assessing the speed of sound, along with density of NaDC and NaC solutions without and with glycylglycine. We have already published information on the operation principle and calibration method of the DSA-5000 device [72]. The uncertainty in the speed of sound is ±0.3 m∙s^−1^, while in density, the uncertainty measurement is ±2 × 10^−3^ g∙cm^−3^. However, all the solutions have been prepared by measuring the weights with the help of a balance (Shimadzu, Kyoto, Japan) having a precision of 0.0001 g.

A simple device called a Man Singh survismeter procured from Spectro Lab Equipments Pvt. Ltd. (New Delhi, India) was used to test the solution’s viscosity. In our earlier work, we detailed the operation principle and calibration method of the survismeter [66]. It was anticipated that viscosity measurements would achieve an accuracy of better than 3 percent in flow time. The uncertainty in viscosity has been found to be ±0.020 mPa.s.

## 4. Conclusions

Both the apparent molar isentropic compressibility and apparent molar volume were calculated using speed of sound and density data for both binary (water +bio-surfactant) and ternary (glycyl glycine+ water +bio-surfactant) systems at various temperatures (298.15–318.15 K). Bio-surfactants exhibit a substantial change in their apparent molar volume and in the compressibility characteristics of the monomer relative to its value in water when glycyl glycine is present. Such a methodical inquiry allowed for the inference of a direct relationship between the aforementioned variables. From $V_{\phi }$ and $\kappa_{s,\phi }$ values, it can be observed that at a lower concentration of bio-surfactants, hydrophilic-hydrophilic interactions are playing their role as both the parameters increase with [bile salt]; however, at higher concentrations of NaC and NaDC, hydrophobic-hydrophobic interactions are major contributors as $V_{\phi }$ and $\kappa_{s,\phi }$ remains almost constant. Higher values of $V_{\phi }$ in the presence of glycyl glycine compared to its absence (pure water) have been seen, and this may be a consequence of an inclusion inside the cavity of the polar head, or it may be a reflection of interactions between the –OH groups of nearby glycyl glycine molecules. However, it is believed that it is possible to obtain a clearer effect in this area of research through a reduction in the relative viscosity of the medium, which is further evidenced by viscosity data managed in this paper.

## Figures and Tables

**Table 1:** Apparent molar volume, $V_{\phi }$/10^−4^(m^3^∙mol^−1^) values for NaC and NaDC in pure water and 0.001, 0.005 and 0.010 mol∙kg^−1^ aqueous solution of glycyl glycine at different temperatures.

NaC						NaDC					
[NaC] mmol∙kg^−1^	293.15 K	298.15 K	303.15 K	308.15 K	313.15 K	[NaDC] mmol∙kg^−1^	293.15 K	298.15 K	303.15 K	308.15 K	313.15 K
[Pure Water]											
**2**	30.43	30.70	30.98	31.21	31.45	**1**	23.76	23.97	24.38	25.10	25.93
**4**	31.50	31.70	32.01	32.17	32.28	**2**	27.07	27.94	28.06	29.20	29.08
**6**	31.84	32.10	32.33	32.45	32.52	**3**	28.84	29.76	29.99	30.53	30.39
**8**	31.85	32.25	32.49	32.57	32.68	**4**	29.90	30.57	30.80	31.14	31.25
**10**	31.91	32.23	32.48	32.58	32.75	**5**	30.57	31.08	31.45	31.65	31.83
**12**	31.99	32.27	32.48	32.58	32.74	**6**	31.09	31.40	31.92	32.04	32.16
**14**	31.97	32.23	32.48	32.59	32.75	**7**	31.28	31.63	32.06	32.21	32.41
**16**	31.99	32.28	32.46	32.64	32.82	**8**	31.42	31.61	32.14	32.24	32.42
**18**	31.95	32.34	32.53	32.68	32.87	**9**	31.35	31.73	32.13	32.43	32.58
**20**	32.00	32.30	32.60	32.70	32.96	**10**	31.45	31.78	32.14	32.45	32.57
**22**	31.98	32.39	32.60	32.79	32.93	**11**	31.38	31.77	32.27	32.52	32.51
**24**	32.06	32.40	32.65	32.80	32.99	**12**	31.50	31.81	32.27	32.54	32.54
**[** **Glycyl glycine** **] = 0.001 mol∙kg^−1^**											
**2**	30.58	30.90	31.13	31.26	31.45	**1**	23.96	24.87	25.49	26.42	28.06
**4**	31.63	32.18	32.26	32.32	32.28	**2**	27.92	28.44	28.82	29.85	31.06
**6**	31.90	32.35	32.51	32.58	32.72	**3**	29.04	29.66	30.33	31.10	31.85
**8**	31.98	32.49	32.58	32.75	32.84	**4**	30.00	30.50	31.33	31.62	32.24
**10**	32.02	32.53	32.58	32.76	32.91	**5**	30.63	31.08	31.69	31.93	32.44
**12**	32.00	32.53	32.63	32.71	32.96	**6**	31.14	31.31	32.02	32.31	32.64
**14**	32.00	32.51	32.63	32.80	32.98	**7**	31.41	31.51	32.19	32.34	32.67
**16**	31.99	32.50	32.58	32.71	33.00	**8**	31.71	31.67	32.28	32.39	32.65
**18**	31.96	32.52	32.62	32.68	32.94	**9**	31.75	31.89	32.42	32.52	32.78
**20**	32.04	32.44	32.65	32.70	32.99	**10**	31.81	32.05	32.54	32.59	32.92
**22**	31.94	32.49	32.63	32.75	32.94	**11**	31.88	32.03	32.58	32.75	33.09
**24**	32.02	32.48	32.60	32.78	32.94	**12**	31.98	32.06	32.57	32.82	33.04
**[Glycyl glycine] = 0.005 mol∙kg^−1^**											
**2**	30.78	30.95	31.23	31.31	31.70	**1**	24.16	25.17	26.70	27.83	28.97
**4**	31.67	32.15	32.36	32.59	32.76	**2**	27.72	28.74	29.62	30.41	31.05
**6**	31.97	32.41	32.63	32.63	32.74	**3**	29.24	30.16	30.49	31.40	32.08
**8**	32.00	32.58	32.65	32.69	32.82	**4**	30.02	30.59	31.40	31.82	32.54
**10**	32.04	32.57	32.63	32.69	32.92	**5**	30.55	31.07	31.93	32.43	32.76
**12**	32.03	32.54	32.62	32.71	32.95	**6**	31.00	31.51	32.26	32.66	33.14
**14**	32.00	32.52	32.61	32.77	32.95	**7**	31.25	31.62	32.60	32.71	33.15
**16**	32.06	32.51	32.64	32.73	32.98	**8**	31.55	32.04	32.64	32.79	33.23
**18**	32.06	32.47	32.61	32.78	32.92	**9**	31.84	32.46	32.79	33.09	33.36
**20**	32.01	32.55	32.60	32.75	32.98	**10**	31.98	32.45	32.98	33.01	33.51
**22**	32.01	32.49	32.57	32.75	32.98	**11**	32.07	32.57	33.05	33.20	33.58
**24**	32.05	32.47	32.63	32.73	32.99	**12**	32.19	32.76	33.31	33.52	33.81
**[Glycyl glycine] = 0.010 mol∙kg^−1^**											
**2**	30.87	31.09	31.27	31.45	31.64	**1**	24.76	25.37	26.89	27.92	29.17
**4**	31.84	32.19	32.50	32.61	32.75	**2**	28.26	29.03	30.16	30.90	31.40
**6**	32.06	32.47	32.75	32.87	33.00	**3**	29.83	30.35	31.19	31.52	32.24
**8**	32.15	32.51	32.72	32.87	33.03	**4**	30.46	30.84	31.57	32.19	32.73
**10**	32.12	32.59	32.77	32.91	33.02	**5**	30.88	31.24	32.06	32.62	32.93
**12**	32.12	32.56	32.80	32.95	33.08	**6**	31.28	31.65	32.40	32.76	33.26
**14**	32.14	32.61	32.90	33.06	33.10	**7**	31.66	31.93	32.71	32.89	33.31
**16**	32.11	32.60	32.95	33.04	33.14	**8**	31.80	32.11	32.81	33.10	33.49
**18**	32.05	32.60	32.87	33.09	33.16	**9**	31.99	32.28	32.99	33.26	33.54
**20**	32.15	32.67	32.96	33.09	33.18	**10**	32.14	32.48	33.09	33.38	33.71
**22**	32.14	32.68	32.95	33.09	33.16	**11**	32.27	32.59	33.23	33.53	33.81
**24**	32.09	32.65	32.93	33.03	33.21	**12**	32.34	32.72	33.37	33.65	33.89

Standard uncertainties, u, are u(T) = 0.01 K, u(molality of NaC) = 0.001 mol·kg^−1^, u(molality of NaDC) = 0.002 mol·kg^−1^ and u(V_Φ_) = 0.05 × 10^−6^ m^3^∙mol^−1^.

**Table 2:** Isentropic compressibility, $\kappa_{s}$ (TPa^−1^) values for NaC and NaDC in pure water and 0.001, 0.005 and 0.010 mol∙kg^−1^ aqueous solution of glycyl glycine at different temperatures.

NaC						NaDC					
[NaC] mmol∙kg^−1^	293.15 K	298.15 K	303.15 K	308.15 K	313.15 K	[NaDC] mmol∙kg^−1^	293.15 K	298.15 K	303.15 K	308.15 K	313.15 K
[Pure water]											
**2**	455.03	447.12	440.49	435.06	430.72	**1**	454.99	447.25	440.68	435.28	430.93
**4**	454.48	446.73	440.17	434.74	430.44	**2**	454.68	447.05	440.45	435.10	430.79
**6**	454.09	446.29	439.76	434.35	430.10	**3**	454.45	446.85	440.29	434.94	430.65
**8**	453.66	445.82	439.35	434.00	429.75	**4**	454.24	446.69	440.14	434.79	430.51
**10**	453.16	445.35	438.95	433.61	429.40	**5**	454.07	446.51	439.96	434.65	430.37
**12**	452.65	444.89	438.55	433.23	429.04	**6**	453.89	446.35	439.85	434.49	430.23
**14**	452.18	444.49	438.14	432.85	428.69	**7**	453.72	446.17	439.66	434.31	430.09
**16**	451.75	444.06	437.78	432.48	428.35	**8**	453.56	445.98	439.47	434.16	429.95
**18**	451.38	443.75	437.47	432.11	428.00	**9**	453.39	445.76	439.32	434.01	429.81
**20**	450.94	443.47	437.13	431.80	427.70	**10**	453.22	445.59	439.14	433.87	429.66
**22**	450.74	443.04	436.78	431.47	427.43	**11**	453.03	445.43	438.97	433.73	429.54
**24**	450.32	442.68	436.42	431.16	427.13	**12**	452.87	445.29	438.85	433.56	429.41
**[Glycyl glycine] = 0.005 mol∙kg^−1^**											
**2**	454.62	446.73	440.10	434.70	430.40	**1**	454.58	446.86	440.26	434.86	430.53
**4**	454.08	446.35	439.77	434.38	430.10	**2**	454.39	446.64	440.07	434.70	430.39
**6**	453.65	445.90	439.37	434.01	429.79	**3**	454.14	446.48	439.93	434.56	430.26
**8**	453.24	445.43	438.98	433.66	429.43	**4**	453.92	446.32	439.78	434.41	430.13
**10**	452.73	444.95	438.57	433.28	429.07	**5**	453.73	446.13	439.61	434.29	429.98
**12**	452.25	444.48	438.16	432.90	428.71	**6**	453.52	445.93	439.45	434.15	429.84
**14**	451.80	444.08	437.77	432.52	428.37	**7**	453.32	445.75	439.32	434.00	429.71
**16**	451.34	443.67	437.37	432.18	428.02	**8**	453.13	445.58	439.14	433.85	429.54
**18**	450.96	443.37	437.12	431.82	427.69	**9**	452.93	445.41	438.99	433.70	429.40
**20**	450.52	443.05	436.78	431.47	427.35	**10**	452.73	445.26	438.85	433.54	429.26
**22**	450.35	442.70	436.41	431.14	427.01	**11**	452.57	445.09	438.70	433.42	429.14
**24**	449.91	442.31	436.06	430.83	426.68	**12**	452.40	444.91	438.55	433.29	429.00
**[Glycyl glycine] = 0.010 mol∙kg^−1^**											
**2**	454.18	446.30	439.67	434.28	430.00	**1**	454.18	446.40	439.81	434.40	430.12
**4**	453.63	445.90	439.34	433.94	429.70	**2**	453.98	446.26	439.65	434.27	429.98
**6**	453.18	445.46	438.93	433.57	429.32	**3**	453.78	446.08	439.48	434.12	429.86
**8**	452.78	445.01	438.55	433.22	429.02	**4**	453.63	445.91	439.34	433.98	429.73
**10**	452.27	444.56	438.15	432.84	428.66	**5**	453.46	445.72	439.17	433.85	429.58
**12**	451.86	444.12	437.75	432.50	428.31	**6**	453.28	445.57	439.01	433.70	429.44
**14**	451.37	443.70	437.36	432.08	427.98	**7**	453.12	445.40	438.88	433.57	429.31
**16**	451.00	443.26	436.96	431.72	427.64	**8**	452.97	445.23	438.70	433.40	429.18
**18**	450.57	442.91	436.69	431.35	427.34	**9**	452.82	445.09	438.56	433.25	429.07
**20**	450.11	442.66	436.33	431.02	427.00	**10**	452.65	444.92	438.40	433.12	428.94
**22**	449.90	442.26	435.94	430.69	426.70	**11**	452.50	444.76	438.26	432.99	428.80
**24**	449.49	441.89	435.65	430.44	426.37	**12**	452.31	444.59	438.11	432.87	428.67

Standard uncertainties, u, are u(T) = 0.01 K, u(molality of NaC) = 0.001 mol·kg^−1^, u(molality of NaDC) = 0.002 mol·kg^−1^ and u(κ_s_) = 0.21 TPa^−1^.

**Table 3:** Apparent molar isentropic compression, $\kappa_{s,\phi }$ /10^−3^ (m^3^∙mol^−1^∙Pa^−1^) values for NaC and NaDC in pure water and in 0.001, 0.005 and 0.010 mol∙kg^−1^ aqueous solution of glycyl glycine at different temperatures.

NaC						NaDC					
[NaC] mmol∙kg^−1^	293.15 K	298.15 K	303.15 K	308.15 K	313.15 K	[NaDC] mmol∙kg^−1^	293.15 K	298.15 K	303.15 K	308.15 K	313.15 K
[Pure Water]											
**2**	−21.46	−16.73	−14.63	−13.29	−11.48	**1**	−63.10	−36.73	−26.78	−20.51	−17.25
**4**	−16.94	−10.74	−8.15	−7.56	−5.65	**2**	−40.38	−21.48	−18.00	−12.33	−8.85
**6**	−12.89	−9.68	−7.38	−6.74	−4.68	**3**	−29.74	−15.95	−12.44	−8.67	−6.01
**8**	−11.46	−9.48	−7.06	−5.98	−4.46	**4**	−23.81	−12.22	−9.41	−6.60	−4.42
**10**	−11.30	−9.47	−6.91	−5.81	−4.22	**5**	−19.35	−10.45	−8.11	−5.35	−3.41
**12**	−11.24	−9.31	−6.74	−5.75	−4.21	**6**	−16.62	−8.99	−6.05	−4.67	−2.69
**14**	−10.93	−8.83	−6.67	−5.62	−4.15	**7**	−14.57	−8.20	−5.84	−4.43	−2.24
**16**	−10.47	−8.66	−6.35	−5.48	−4.01	**8**	−12.89	−7.78	−5.71	−4.06	−1.99
**18**	−9.78	−7.81	−5.75	−5.34	−3.93	**9**	−11.80	−7.79	−5.28	−3.62	−1.73
**20**	−9.56	−7.03	−5.47	−4.98	−3.64	**10**	−10.92	−7.21	−5.09	−3.27	−1.64
**22**	−8.32	−6.99	−5.30	−4.70	−3.28	**11**	−10.34	−6.79	−4.89	−2.94	−1.35
**24**	−8.17	−6.74	−5.12	−4.45	−3.09	**12**	−9.59	−6.17	−4.28	−2.93	−1.23
**[Glycyl glycine] = 0.005 mol∙kg^−1^**											
**2**	−19.88	−15.88	−14.09	−12.89	−10.11	**1**	−56.48	−30.80	−24.11	−21.55	−19.14
**4**	−16.13	−10.01	−7.97	−7.17	−5.36	**2**	−30.82	−19.22	−14.50	−11.54	−9.40
**6**	−12.94	−9.18	−7.19	−6.37	−4.20	**3**	−24.01	−13.16	−9.83	−7.51	−5.68
**8**	−11.22	−9.10	−6.76	−5.64	−4.17	**4**	−19.82	−10.38	−7.37	−5.85	−3.86
**10**	−11.21	−9.26	−6.63	−5.47	−4.11	**5**	−16.75	−9.23	−6.26	−4.08	−3.16
**12**	−10.93	−9.24	−6.60	−5.38	−4.06	**6**	−14.83	−8.46	−5.34	−3.35	−2.50
**14**	−10.53	−8.72	−6.42	−5.28	−3.92	**7**	−13.52	−7.75	−4.34	−3.00	−2.08
**16**	−10.30	−8.43	−6.39	−5.06	−3.85	**8**	−12.28	−6.99	−4.23	−2.70	−2.13
**18**	−9.66	−7.61	−5.50	−4.92	−3.75	**9**	−11.40	−6.32	−3.77	−2.39	−1.80
**20**	−9.50	−6.97	−5.25	−4.81	−3.67	**10**	−10.78	−5.85	−3.35	−2.31	−1.50
**22**	−8.11	−6.69	−5.20	−4.57	−3.61	**11**	−9.96	−5.46	−3.01	−1.86	−1.19
**24**	−8.05	−6.58	−5.00	−4.33	−3.52	**12**	−9.24	−5.20	−2.71	−1.40	−0.93
**[Glycyl glycine] = 0.010 mol∙kg^−1^**											
**2**	−18.21	−14.51	−13.43	−11.58	−9.84	**1**	−49.70	−32.35	−24.46	−21.99	−18.92
**4**	−15.63	−9.94	−7.67	−6.99	−5.21	**2**	−27.50	−15.93	−12.81	−10.50	−9.07
**6**	−12.92	−9.09	−7.05	−6.13	−5.18	**3**	−20.15	−11.56	−9.38	−7.18	−5.33
**8**	−11.13	−8.90	−6.51	−5.39	−4.11	**4**	−15.23	−9.39	−7.04	−5.36	−3.67
**10**	−11.04	−8.68	−6.37	−5.25	−4.06	**5**	−12.61	−8.33	−6.11	−3.89	−3.00
**12**	−10.25	−8.50	−6.22	−4.91	−3.97	**6**	−10.97	−7.01	−5.19	−3.23	−2.28
**14**	−10.19	−8.26	−6.04	−5.12	−3.73	**7**	−9.53	−6.26	−4.20	−2.66	−1.76
**16**	−9.50	−8.17	−6.02	−4.95	−3.62	**8**	−8.34	−5.79	−4.12	−2.49	−1.33
**18**	−9.23	−7.59	−5.28	−4.88	−3.34	**9**	−7.36	−5.06	−3.48	−2.29	−0.87
**20**	−9.13	−6.62	−5.12	−4.66	−3.30	**10**	−6.87	−4.72	−3.29	−1.86	−0.57
**22**	−7.96	−6.55	−5.10	−4.45	−3.12	**11**	−6.24	−4.38	−2.87	−1.56	−0.46
**24**	−7.84	−6.39	−4.71	−3.98	−3.03	**12**	−6.06	−4.17	−2.59	−1.17	−0.23

Standard uncertainties, u, are u(T) = 0.01 K, u(molality of NaC) = 0.001 mol·kg^−1^, u(molality of NaDC) = 0.002 mol·kg^−1^, and u(κ_s,Φ_) = 0.05 × 10^−3^ m^3^∙mol^−1^∙TPa^−1^.

**Table 4:** Relative viscosity, $\eta_{r}$ values for NaC and NaDC in pure water and in 0.001, 0.005 and 0.010 mol∙kg^−1^ aqueous solution of glycyl glycine at different temperatures.

NaC						NaDC					
[NaC] mmol∙kg^−1^	293.15 K	298.15 K	303.15 K	308.15 K	313.15 K	[NaDC] mmol∙kg^−1^	293.15 K	298.15 K	303.15 K	308.15 K	313.15 K
[Pure water]											
**2**	1.002	1.003	1.004	1.004	1.004	**1**	1.001	1.001	1.002	1.002	1.003
**4**	1.005	1.006	1.007	1.008	1.008	**2**	1.003	1.003	1.003	1.004	1.004
**6**	1.009	1.010	1.011	1.011	1.012	**3**	1.004	1.004	1.005	1.005	1.006
**8**	1.014	1.014	1.015	1.016	1.017	**4**	1.007	1.007	1.008	1.009	1.010
**10**	1.019	1.018	1.020	1.020	1.020	**5**	1.012	1.013	1.015	1.016	1.018
**12**	1.023	1.024	1.025	1.025	1.026	**6**	1.025	1.028	1.031	1.034	1.038
**14**	1.030	1.031	1.032	1.033	1.034	**7**	1.034	1.038	1.041	1.046	1.051
**16**	1.035	1.036	1.038	1.039	1.041	**8**	1.038	1.043	1.046	1.052	1.057
**18**	1.038	1.040	1.042	1.045	1.048	**9**	1.043	1.047	1.053	1.057	1.063
**20**	1.042	1.044	1.047	1.050	1.052	**10**	1.045	1.049	1.055	1.061	1.065
**22**	1.046	1.048	1.051	1.054	1.056	**11**	1.048	1.052	1.058	1.063	1.069
**24**	1.049	1.052	1.055	1.057	1.059	**12**	1.049	1.054	1.061	1.066	1.071
**[Glycyl glycine] = 0.001 mol∙kg^−1^**											
**2**	1.006	1.007	1.007	1.008	1.010	**1**	1.005	1.005	1.006	1.006	1.007
**4**	1.009	1.011	1.012	1.013	1.014	**2**	1.006	1.007	1.007	1.008	1.008
**6**	1.013	1.014	1.016	1.016	1.018	**3**	1.008	1.008	1.008	1.009	1.010
**8**	1.017	1.018	1.019	1.021	1.022	**4**	1.010	1.011	1.012	1.012	1.014
**10**	1.022	1.023	1.025	1.027	1.028	**5**	1.013	1.017	1.019	1.019	1.022
**12**	1.028	1.029	1.031	1.032	1.034	**6**	1.026	1.031	1.035	1.037	1.047
**14**	1.035	1.036	1.039	1.041	1.042	**7**	1.035	1.041	1.045	1.049	1.054
**16**	1.040	1.041	1.044	1.046	1.049	**8**	1.039	1.047	1.050	1.055	1.060
**18**	1.043	1.045	1.048	1.052	1.054	**9**	1.043	1.051	1.056	1.061	1.066
**20**	1.047	1.049	1.054	1.056	1.059	**10**	1.046	1.053	1.059	1.064	1.069
**22**	1.051	1.053	1.057	1.061	1.063	**11**	1.048	1.056	1.061	1.067	1.073
**24**	1.054	1.057	1.061	1.064	1.067	**12**	1.049	1.057	1.065	1.069	1.074
**[Glycyl glycine] = 0.005 mol∙kg^−1^**											
**2**	1.006	1.007	1.007	1.009	1.010	**1**	1.001	1.002	1.003	1.003	1.003
**4**	1.009	1.011	1.012	1.013	1.014	**2**	1.003	1.004	1.004	1.004	1.005
**6**	1.013	1.014	1.016	1.017	1.018	**3**	1.005	1.005	1.005	1.006	1.006
**8**	1.017	1.019	1.020	1.021	1.022	**4**	1.007	1.008	1.009	1.009	1.010
**10**	1.022	1.024	1.026	1.027	1.028	**5**	1.010	1.014	1.016	1.016	1.018
**12**	1.028	1.030	1.031	1.033	1.034	**6**	1.023	1.028	1.032	1.034	1.043
**14**	1.035	1.037	1.039	1.041	1.042	**7**	1.032	1.038	1.042	1.046	1.050
**16**	1.040	1.042	1.044	1.046	1.048	**8**	1.037	1.043	1.047	1.052	1.056
**18**	1.043	1.046	1.049	1.052	1.055	**9**	1.040	1.048	1.052	1.057	1.062
**20**	1.047	1.050	1.054	1.056	1.060	**10**	1.043	1.050	1.055	1.060	1.065
**22**	1.051	1.054	1.058	1.061	1.064	**11**	1.045	1.053	1.058	1.063	1.068
**24**	1.054	1.059	1.061	1.064	1.068	**12**	1.046	1.054	1.061	1.066	1.072
**[Glycyl glycine] = 0.010 mol∙kg^−1^**											
**2**	1.007	1.007	1.007	1.009	1.010	**1**	1.002	1.002	1.003	1.003	1.003
**4**	1.009	1.011	1.011	1.013	1.014	**2**	1.003	1.004	1.004	1.005	1.005
**6**	1.013	1.015	1.016	1.018	1.018	**3**	1.004	1.005	1.006	1.006	1.007
**8**	1.017	1.020	1.019	1.022	1.022	**4**	1.007	1.008	1.009	1.010	1.011
**10**	1.022	1.025	1.025	1.028	1.028	**5**	1.010	1.014	1.016	1.016	1.018
**12**	1.028	1.030	1.031	1.033	1.034	**6**	1.020	1.028	1.032	1.034	1.043
**14**	1.035	1.037	1.039	1.042	1.042	**7**	1.032	1.039	1.042	1.046	1.050
**16**	1.040	1.042	1.044	1.046	1.049	**8**	1.037	1.043	1.047	1.052	1.056
**18**	1.043	1.046	1.048	1.052	1.055	**9**	1.040	1.047	1.052	1.057	1.062
**20**	1.047	1.050	1.053	1.056	1.061	**10**	1.042	1.050	1.055	1.060	1.065
**22**	1.051	1.054	1.057	1.061	1.065	**11**	1.045	1.053	1.058	1.063	1.068
**24**	1.054	1.059	1.061	1.065	1.069	**12**	1.046	1.054	1.061	1.065	1.072

Standard uncertainties, u, are u(T) = 0.01 K, u(molality of NaC) = 0.001 mol·kg^−1^, u(molality of NaDC) = 0.002 mol·kg^−1^, and u(η_r_) = 0.020 mPa∙s^−1^.

**Table 5:** Viscous relaxation time, τ (ps) values for NaC and NaDC in pure water and in 0.001, 0.005 and 0.010 mol∙kg^−1^ aqueous solution of glycyl glycine at different temperatures.

NaC						NaDC					
[NaC] mmol∙kg^−1^	293.15 K	298.15 K	303.15 K	308.15 K	313.15 K	[NaDC] mmol∙kg^−1^	293.15 K	298.15 K	303.15 K	308.15 K	313.15 K
[Pure Water]											
**2**	0.609	0.532	0.470	0.419	0.376	**1**	0.609	0.532	0.469	0.418	0.376
**4**	0.610	0.534	0.471	0.420	0.378	**2**	0.609	0.532	0.470	0.419	0.376
**6**	0.612	0.535	0.472	0.421	0.379	**3**	0.609	0.532	0.471	0.419	0.377
**8**	0.615	0.537	0.474	0.423	0.380	**4**	0.611	0.534	0.472	0.421	0.378
**10**	0.617	0.538	0.476	0.424	0.381	**5**	0.614	0.537	0.475	0.423	0.381
**12**	0.618	0.541	0.478	0.426	0.383	**6**	0.622	0.545	0.482	0.431	0.389
**14**	0.622	0.544	0.481	0.428	0.386	**7**	0.627	0.550	0.487	0.436	0.393
**16**	0.625	0.546	0.483	0.431	0.388	**8**	0.629	0.552	0.489	0.438	0.395
**18**	0.626	0.548	0.485	0.433	0.390	**9**	0.632	0.554	0.492	0.440	0.397
**20**	0.628	0.550	0.487	0.434	0.391	**10**	0.633	0.555	0.493	0.441	0.398
**22**	0.630	0.551	0.488	0.436	0.393	**11**	0.634	0.556	0.494	0.442	0.400
**24**	0.631	0.553	0.490	0.437	0.394	**12**	0.635	0.557	0.495	0.443	0.400
**[Glycyl glycine] = 0.001 mol∙kg^−1^**											
**2**	0.614	0.535	0.473	0.422	0.379	**1**	0.613	0.534	0.472	0.421	0.378
**4**	0.615	0.536	0.474	0.423	0.381	**2**	0.614	0.535	0.473	0.422	0.379
**6**	0.617	0.538	0.476	0.425	0.382	**3**	0.614	0.535	0.473	0.422	0.379
**8**	0.618	0.539	0.477	0.426	0.383	**4**	0.615	0.536	0.475	0.423	0.381
**10**	0.621	0.541	0.479	0.428	0.385	**5**	0.617	0.539	0.477	0.426	0.384
**12**	0.624	0.544	0.482	0.430	0.387	**6**	0.624	0.547	0.485	0.433	0.393
**14**	0.627	0.547	0.485	0.433	0.390	**7**	0.630	0.552	0.489	0.438	0.396
**16**	0.630	0.549	0.487	0.435	0.392	**8**	0.631	0.554	0.492	0.441	0.398
**18**	0.631	0.551	0.489	0.437	0.394	**9**	0.634	0.557	0.494	0.443	0.400
**20**	0.633	0.553	0.491	0.439	0.395	**10**	0.635	0.557	0.495	0.444	0.401
**22**	0.635	0.554	0.492	0.440	0.396	**11**	0.636	0.559	0.496	0.445	0.402
**24**	0.636	0.556	0.493	0.441	0.398	**12**	0.637	0.559	0.498	0.446	0.402
**[Glycyl glycine] = 0.005 mol∙kg^−1^**											
**2**	0.616	0.535	0.474	0.424	0.380	**1**	0.613	0.533	0.471	0.421	0.378
**4**	0.617	0.537	0.475	0.425	0.382	**2**	0.614	0.534	0.472	0.422	0.378
**6**	0.619	0.538	0.477	0.426	0.383	**3**	0.614	0.534	0.472	0.422	0.379
**8**	0.621	0.540	0.478	0.428	0.384	**4**	0.615	0.535	0.474	0.423	0.380
**10**	0.623	0.542	0.480	0.430	0.386	**5**	0.617	0.538	0.477	0.426	0.383
**12**	0.626	0.545	0.483	0.432	0.388	**6**	0.625	0.546	0.484	0.433	0.393
**14**	0.630	0.548	0.486	0.435	0.391	**7**	0.630	0.551	0.489	0.438	0.395
**16**	0.632	0.550	0.488	0.437	0.393	**8**	0.632	0.553	0.491	0.441	0.397
**18**	0.634	0.552	0.490	0.439	0.395	**9**	0.634	0.555	0.493	0.443	0.399
**20**	0.635	0.554	0.492	0.440	0.397	**10**	0.636	0.556	0.495	0.444	0.400
**22**	0.637	0.555	0.493	0.442	0.398	**11**	0.637	0.558	0.496	0.445	0.401
**24**	0.639	0.557	0.494	0.443	0.399	**12**	0.637	0.558	0.497	0.446	0.403
**[Glycyl glycine] = 0.010 mol∙kg^−1^**											
**2**	0.618	0.536	0.475	0.425	0.381	**1**	0.615	0.534	0.473	0.423	0.379
**4**	0.619	0.538	0.476	0.427	0.383	**2**	0.615	0.534	0.473	0.423	0.380
**6**	0.621	0.539	0.478	0.428	0.384	**3**	0.616	0.535	0.474	0.424	0.380
**8**	0.622	0.541	0.479	0.429	0.385	**4**	0.617	0.536	0.475	0.425	0.382
**10**	0.625	0.544	0.481	0.432	0.387	**5**	0.619	0.539	0.478	0.428	0.384
**12**	0.628	0.546	0.484	0.434	0.389	**6**	0.625	0.547	0.486	0.435	0.394
**14**	0.631	0.549	0.487	0.437	0.392	**7**	0.632	0.552	0.490	0.440	0.396
**16**	0.634	0.551	0.489	0.438	0.394	**8**	0.635	0.554	0.492	0.442	0.398
**18**	0.635	0.553	0.491	0.440	0.396	**9**	0.637	0.556	0.495	0.444	0.400
**20**	0.637	0.555	0.493	0.442	0.398	**10**	0.638	0.557	0.496	0.445	0.401
**22**	0.639	0.556	0.494	0.443	0.399	**11**	0.639	0.558	0.497	0.446	0.402
**24**	0.640	0.558	0.495	0.445	0.400	**12**	0.640	0.559	0.498	0.447	0.404

Standard uncertainties, u, are u(T) = 0.01 K, u(molality of NaC) = 0.001 mol·kg^−1^, u(molality of NaDC) = 0.002 mol·kg^−1^, and u(τ) = 0.01 × 10^−3^ ps.

**Table 6:** Specification of chemicals used.

Chemical Name	Source	CAS No.	Mol.Wt./kg∙mol^−1^	Purification Method	Mass Fraction Purity ^a^
Glycyl glycine	Spectrochem Pvt. Ltd.	556–50–3	0.132	None	0.98
Sodium cholate	Himedia Pvt. Ltd.	361–09–1	0.431	Recrystallization	0.98
Sodium deoxycholate	Himedia Pvt. Ltd.	302–95–4	0.415	Recrystallization	0.98

^a^ Declared by the supplier.

## Data Availability

Additional data is provided in Supplementary Materials.

## Supplementary Materials

The following supporting information can be downloaded at: https://www.mdpi.com/article/10.3390/molecules27248998/s1, Table S1: Density, (kg∙m^−3^) values for NaC and NaDC in pure water and 0.001, 0.005 and 0.010 mol∙kg^−1^ aqueous solution of glycyl dipeptide at different temperatures.; Table S2: Speed of sound, (m∙s^−1^) values for NaC and NaDC in pure water and 0.001, 0.005 and 0.010 mol∙kg^−1^ aqueous solution of glycyl dipeptide at different temperatures.; Table S3: Viscosity, (mPa∙s) values for NaC and NaDC in pure water and 0.001, 0.005 and 0.010 mol∙kg^−1^ aqueous solution of glycyl dipeptide at different temperatures.

## Sample Availability

Samples are available from the authors.
