# Supplementary material for: Volumetric, Compressibility and Viscometric Approach to Study the Interactional Behaviour of Sodium Cholate and Sodium Deoxycholate in Aqueous Glycyl Glycine
Source: Molecules. 2022 Dec 16;27(24):8998. doi: 10.3390/molecules27248998 (PMC9782853; doi:10.3390/molecules27248998)
Supplement: Supplementary file 1 [file molecules-27-08998-s001.zip › molecules-2063344-supplementary.pdf]

Supplementary Material

Volumetric, Compressibility and Viscometric Approach to Study the Interactional Behaviour of Sodium Cholate and Sodium Deoxycholate in Aqueous Glycyl Glycine

Table S1: Density, (kg·m^{-3}) values for NaC and NaDC in pure water and in 0.001, 0.005 and 0.010 mol·kg^{-1} aqueous solution of glycyl dipeptide at different temperaturesφ

NaC						NaDC					
**[NaC] mmol·kg^{-1}**	**293.15 K**	**298.15 K**	**303.15 K**	**308.15 K**	**313.15 K**	**[NaDC] mmol·kg^{-1}**	**293.15 K**	**298.15 K**	**303.15 K**	**308.15 K**	**313.15 K**
**Pure Water**											
**2**	998.46	997.29	995.88	994.26	992.43	**1**	998.38	997.22	995.81	994.18	992.35
**4**	998.67	997.50	996.08	994.46	992.63	**2**	998.49	997.31	995.91	994.27	992.45
**6**	998.88	997.70	996.29	994.66	992.84	**3**	998.59	997.39	995.98	994.35	992.53
**8**	999.10	997.91	996.49	994.87	993.04	**4**	998.67	997.48	996.07	994.44	992.61
**10**	999.32	998.13	996.70	995.08	993.24	**5**	998.75	997.56	996.14	994.52	992.69
**12**	999.53	998.34	996.91	995.29	993.45	**6**	998.83	997.65	996.22	994.59	992.76
**14**	999.76	998.56	997.13	995.50	993.66	**7**	998.92	997.73	996.30	994.68	992.84
**16**	999.97	998.77	997.34	995.70	993.85	**8**	999.01	997.83	996.39	994.77	992.93
**18**	1000.20	998.97	997.54	995.90	994.05	**9**	999.12	998.01	996.48	994.84	993.01
**20**	1000.41	999.19	997.74	996.10	994.24	**10**	999.21	998.11	996.58	994.93	993.10
**22**	1000.63	999.39	997.94	996.29	994.45	**11**	999.32	998.20	996.66	995.02	993.20
**24**	1000.83	999.60	998.14	996.49	994.64	**12**	999.40	998.20	996.75	995.10	993.28
**[Glycyl Dipeptide] = 0.001 mol·kg^{-1}**											
**2**	998.48	997.32	995.84	994.31	992.49	**1**	998.44	997.28	995.79	994.26	992.43
**4**	998.69	997.51	996.03	994.50	992.70	**2**	998.54	997.37	995.89	994.34	992.51
**6**	998.90	997.72	996.24	994.70	992.89	**3**	998.64	997.47	995.97	994.42	992.59
**8**	999.12	997.92	996.44	994.90	993.09	**4**	998.72	997.55	996.04	994.50	992.67

**10**	999.33	998.13	996.65	995.11	993.29	**5**	998.81	997.63	996.13	994.59	992.75
**12**	999.56	998.34	996.86	995.32	993.49	**6**	998.89	997.72	996.21	994.66	992.83
**14**	999.78	998.55	997.06	995.52	993.69	**7**	998.97	997.81	996.29	994.75	992.92
**16**	1000.00	998.77	997.28	995.74	993.89	**8**	999.05	997.90	996.37	994.84	993.01
**18**	1000.22	998.97	997.48	995.95	994.10	**9**	999.14	997.98	996.45	994.92	993.09
**20**	1000.43	999.20	997.69	996.15	994.29	**10**	999.23	998.06	996.53	995.00	993.16
**22**	1000.67	999.40	997.90	996.35	994.51	**11**	999.32	998.15	996.62	995.08	993.23
**24**	1000.87	999.61	998.11	996.55	994.71	**12**	999.40	998.24	996.71	995.15	993.32
**[Glycyl Dipeptide] = 0.005 mol·kg^{-1}**											
**2**	998.69	997.52	996.11	994.48	992.65	**1**	998.65	997.48	996.05	994.42	992.58
**4**	998.90	997.71	996.30	994.67	992.83	**2**	998.76	997.57	996.14	994.50	992.66
**6**	999.11	997.92	996.50	994.88	993.04	**3**	998.85	997.65	996.24	994.59	992.74
**8**	999.33	998.12	996.71	995.08	993.25	**4**	998.94	997.75	996.31	994.67	992.82
**10**	999.55	998.33	996.92	995.29	993.44	**5**	999.03	997.83	996.38	994.74	992.90
**12**	999.77	998.54	997.13	995.50	993.64	**6**	999.11	997.91	996.46	994.82	992.96
**14**	999.99	998.75	997.34	995.69	993.85	**7**	999.20	998.00	996.53	994.90	993.05
**16**	1000.20	998.96	997.54	995.91	994.05	**8**	999.27	998.07	996.62	994.98	993.12
**18**	1000.42	999.18	997.75	996.11	994.26	**9**	999.35	998.13	996.69	995.04	993.20
**20**	1000.65	999.38	997.96	996.32	994.45	**10**	999.43	998.22	996.76	995.14	993.26
**22**	1000.87	999.60	998.18	996.52	994.66	**11**	999.51	998.29	996.84	995.20	993.34
**24**	1001.07	999.81	998.37	996.73	994.85	**12**	999.59	998.36	996.89	995.25	993.39
**[Glycyl Dipeptide] = 0.010 mol·kg^{-1}**											
**2**	999.12	998.02	996.64	995.02	993.20	**1**	999.08	997.98	996.59	994.96	993.13
**4**	999.32	998.22	996.83	995.21	993.39	**2**	999.18	998.07	996.67	995.04	993.21
**6**	999.54	998.42	997.03	995.41	993.58	**3**	999.26	998.15	996.75	995.13	993.29
**8**	999.75	998.63	997.24	995.61	993.78	**4**	999.35	998.25	996.84	995.20	993.36
**10**	999.97	998.83	997.44	995.81	993.99	**5**	999.44	998.33	996.92	995.27	993.44
**12**	1000.19	999.04	997.64	996.01	994.18	**6**	999.52	998.41	996.99	995.35	993.51

**14**	1000.40	999.24	997.83	996.20	994.38	**7**	999.60	998.49	996.99	995.43	993.59
**16**	1000.62	999.45	998.03	996.40	994.57	**8**	999.69	998.57	997.06	995.50	993.66
**18**	1000.85	999.66	998.24	996.59	994.77	**9**	999.77	998.65	997.14	995.57	993.73
**20**	1001.05	999.86	998.43	996.79	994.97	**10**	999.84	998.72	997.21	995.64	993.80
**22**	1001.26	1000.06	998.63	996.99	995.17	**11**	999.92	998.80	997.29	995.71	993.87
**24**	1001.49	1000.27	998.84	997.20	995.35	**12**	1000.01	998.87	997.35	995.78	993.93

Table S2: Speed of sound, u (m·s^{-1}) values for NaC and NaDC in pure water and 0.001, 0.005 and 0.010 mol·kg^{-1} aqueous solution of glycyl dipeptide at different temperatures.

**NaC**						**NaDC**					
**[NaC] mmol·kg^{-1}**	**293.15 K**	**298.15 K**	**303.15 K**	**308.15 K**	**313.15 K**	**[NaDC] mmol·kg^{-1}**	**293.15 K**	**298.15 K**	**303.15 K**	**308.15 K**	**313.15 K**
**[Pure Water]**											
**2**	1483.60	1497.54	1509.83	1520.46	1529.51	**1**	1483.71	1497.37	1509.56	1520.13	1529.19
**4**	1484.33	1498.03	1510.23	1520.87	1529.85	**2**	1484.14	1497.64	1509.76	1520.39	1529.37
**6**	1484.81	1498.62	1510.77	1521.39	1530.29	**3**	1484.45	1497.91	1509.98	1520.60	1529.56
**8**	1485.35	1499.25	1511.32	1521.86	1530.77	**4**	1484.73	1498.11	1510.18	1520.79	1529.75
**10**	1486.01	1499.88	1511.86	1522.37	1531.23	**5**	1484.94	1498.35	1510.42	1521.21	1529.94
**12**	1486.69	1500.49	1512.39	1522.89	1531.71	**6**	1485.18	1498.56	1510.67	1521.45	1530.12
**14**	1487.29	1501.00	1512.93	1523.39	1532.18	**7**	1485.39	1498.80	1510.93	1521.65	1530.31
**16**	1487.84	1501.58	1513.39	1523.89	1532.64	**8**	1485.58	1499.04	1511.19	1522.03	1530.49
**18**	1488.28	1501.95	1513.77	1524.38	1533.11	**9**	1485.78	1499.28	1511.39	1522.21	1530.68
**20**	1488.85	1502.26	1514.22	1524.78	1533.51	**10**	1486.00	1499.48	1511.62	1522.44	1530.87
**22**	1489.02	1502.83	1514.67	1525.21	1533.83	**11**	1486.22	1499.69	1511.86	1522.21	1531.01
**24**	1489.57	1503.29	1515.13	1525.61	1534.23	**12**	1486.42	1499.92	1511.99	1522.44	1531.19
**[Glycyl Dipeptide] = 0.001 mol·kg^{-1}**											
**2**	1483.75	1497.75	1510.03	1520.64	1529.66	**1**	1483.91	1497.54	1509.80	1520.46	1529.48
**4**	1484.57	1498.26	1510.44	1521.04	1530.00	**2**	1484.20	1497.85	1510.04	1520.69	1529.65
**6**	1485.09	1498.83	1510.98	1521.57	1530.43	**3**	1484.52	1498.09	1510.33	1520.88	1529.84
**8**	1485.60	1499.48	1511.51	1522.05	1530.94	**4**	1484.83	1498.29	1510.54	1521.09	1530.00
**10**	1486.27	1500.12	1512.06	1522.56	1531.42	**5**	1485.09	1498.55	1510.77	1521.28	1530.19

**12**	1486.90	1500.71	1512.60	1523.06	1531.91	**6**	1485.35	1498.78	1511.00	1521.49	1530.38
**14**	1487.51	1501.26	1513.13	1523.59	1532.38	**7**	1485.57	1499.01	1511.19	1521.69	1530.58
**16**	1488.07	1501.84	1513.66	1524.05	1532.83	**8**	1485.85	1499.29	1511.43	1521.91	1530.80
**18**	1488.51	1502.19	1513.97	1524.53	1533.28	**9**	1486.11	1499.56	1511.65	1522.12	1530.96
**20**	1489.10	1502.52	1514.40	1524.96	1533.66	**10**	1486.32	1499.80	1511.86	1522.33	1531.15
**22**	1489.20	1503.03	1514.86	1525.35	1533.94	**11**	1486.53	1500.03	1512.06	1522.52	1531.32
**24**	1489.80	1503.50	1515.30	1525.78	1534.35	**12**	1486.76	1500.20	1512.27	1522.71	1531.50
**[Glycyl Dipeptide] = 0.005 mol·kg^{-1}**											
**2**	1484.09	1498.02	1510.32	1520.91	1529.90	**1**	1484.18	1497.83	1510.09	1520.69	1529.74
**4**	1484.82	1498.51	1510.74	1521.33	1530.29	**2**	1484.42	1498.13	1510.35	1520.90	1529.92
**6**	1485.36	1499.10	1511.28	1521.83	1530.69	**3**	1484.76	1498.33	1510.53	1521.08	1530.09
**8**	1485.87	1499.74	1511.80	1522.29	1531.18	**4**	1485.05	1498.53	1510.73	1521.28	1530.26
**10**	1486.55	1500.40	1512.34	1522.79	1531.67	**5**	1485.30	1498.79	1510.96	1521.44	1530.46
**12**	1487.17	1501.03	1512.89	1523.30	1532.15	**6**	1485.57	1499.06	1511.17	1521.63	1530.66
**14**	1487.74	1501.54	1513.40	1523.81	1532.60	**7**	1485.84	1499.29	1511.35	1521.83	1530.84
**16**	1488.35	1502.08	1513.95	1524.26	1533.07	**8**	1486.09	1499.53	1511.59	1522.03	1531.08
**18**	1488.81	1502.43	1514.22	1524.74	1533.50	**9**	1486.36	1499.77	1511.79	1522.25	1531.27
**20**	1489.37	1502.82	1514.65	1525.20	1533.96	**10**	1486.63	1499.97	1511.99	1522.45	1531.46
**22**	1489.49	1503.26	1515.13	1525.61	1534.41	**11**	1486.84	1500.19	1512.18	1522.62	1531.63
**24**	1490.06	1503.76	1515.58	1526.00	1534.85	**12**	1487.05	1500.44	1512.40	1522.80	1531.83
**[Glycyl Dipeptide] = 0.010 mol·kg^{-1}**											
**2**	1484.48	1498.35	1510.66	1521.24	1530.19	**1**	1484.52	1498.23	1510.46	1521.07	1530.04
**4**	1485.24	1498.88	1511.09	1521.69	1530.58	**2**	1485.04	1498.63	1510.67	1521.25	1530.22
**6**	1485.81	1499.47	1511.64	1522.20	1531.12	**3**	1485.22	1498.85	1510.90	1521.44	1530.38
**8**	1486.32	1500.08	1512.14	1522.65	1531.50	**4**	1485.43	1499.10	1511.08	1521.64	1530.56
**10**	1486.98	1500.68	1512.68	1523.16	1531.98	**5**	1485.66	1499.30	1511.32	1521.81	1530.76
**12**	1487.50	1501.26	1513.21	1523.62	1532.46	**6**	1485.66	1499.30	1511.53	1522.00	1530.95
**14**	1488.14	1501.83	1513.74	1524.21	1532.89	**7**	1485.87	1499.52	1511.76	1522.18	1531.12

**16**	1488.60	1502.41	1514.29	1524.68	1533.35	**8**	1486.05	1499.75	1512.02	1522.41	1531.30
**18**	1489.13	1502.84	1514.59	1525.19	1533.74	**9**	1486.23	1499.93	1512.19	1522.63	1531.45
**20**	1489.74	1503.12	1515.08	1525.63	1534.20	**10**	1486.46	1500.16	1512.42	1522.80	1531.63
**22**	1489.93	1503.65	1515.59	1526.06	1534.59	**11**	1486.65	1500.37	1512.60	1522.99	1531.83
**24**	1490.44	1504.13	1515.94	1526.34	1535.03	**12**	1486.90	1500.60	1512.80	1523.15	1532.00

Table S3: Viscosity, η (mPa·s) values for NaC and NaDC in Pure water and in 0.001, 0.005 and 0.010 mol·kg^{-1} aqueous solution of glycyl dipeptide at different temperatures.

NaC						NaDC					
**[NaC] mmol·kg^{-1}**	**293.15 K**	**298.15 K**	**303.15 K**	**308.15 K**	**313.15 K**	**[NaDC] mmol·kg^{-1}**	**293.15 K**	**298.15 K**	**303.15 K**	**308.15 K**	**313.15 K**
**[Pure Water]**											
**2**	1.004	0.893	0.800	0.722	0.655	**1**	1.003	0.892	0.799	0.720	0.654
**4**	1.007	0.896	0.803	0.725	0.658	**2**	1.005	0.893	0.800	0.722	0.655
**6**	1.011	0.899	0.806	0.727	0.660	**3**	1.006	0.894	0.801	0.723	0.657
**8**	1.016	0.903	0.809	0.730	0.664	**4**	1.009	0.897	0.804	0.725	0.659
**10**	1.021	0.906	0.813	0.733	0.666	**5**	1.014	0.902	0.809	0.730	0.665
**12**	1.025	0.911	0.817	0.737	0.670	**6**	1.027	0.915	0.822	0.743	0.678
**14**	1.032	0.918	0.823	0.742	0.675	**7**	1.036	0.924	0.830	0.752	0.686
**16**	1.037	0.923	0.827	0.747	0.679	**8**	1.040	0.928	0.834	0.756	0.689
**18**	1.040	0.926	0.831	0.751	0.684	**9**	1.045	0.932	0.839	0.760	0.694
**20**	1.044	0.930	0.835	0.755	0.686	**10**	1.047	0.934	0.841	0.763	0.695
**22**	1.048	0.933	0.838	0.758	0.689	**11**	1.050	0.937	0.843	0.764	0.698
**24**	1.051	0.936	0.841	0.760	0.691	**12**	1.051	0.938	0.846	0.766	0.699
**[Glycyl Dipeptide] = 0.001 mol·kg^{-1}**											
**2**	1.012	0.897	0.805	0.728	0.661	**1**	1.011	0.896	0.804	0.726	0.659
**4**	1.015	0.901	0.808	0.731	0.664	**2**	1.012	0.897	0.805	0.727	0.660
**6**	1.019	0.904	0.812	0.734	0.666	**3**	1.013	0.898	0.806	0.728	0.661
**8**	1.023	0.908	0.814	0.737	0.669	**4**	1.016	0.901	0.809	0.731	0.664
**10**	1.028	0.912	0.819	0.741	0.673	**5**	1.019	0.906	0.814	0.736	0.669
**12**	1.034	0.917	0.824	0.745	0.677	**6**	1.032	0.919	0.827	0.748	0.686

**14**	1.041	0.924	0.830	0.751	0.682	**7**	1.041	0.928	0.835	0.757	0.690
**16**	1.046	0.928	0.834	0.755	0.686	**8**	1.045	0.933	0.839	0.762	0.694
**18**	1.049	0.932	0.838	0.759	0.690	**9**	1.049	0.937	0.844	0.765	0.698
**20**	1.053	0.935	0.842	0.762	0.693	**10**	1.052	0.938	0.846	0.768	0.700
**22**	1.057	0.939	0.845	0.766	0.696	**11**	1.054	0.941	0.848	0.770	0.702
**24**	1.060	0.942	0.848	0.768	0.698	**12**	1.055	0.942	0.851	0.772	0.703
**[Glycyl Dipeptide] = 0.005 mol·kg^{-1}**											
**2**	1.016	0.899	0.807	0.731	0.663	**1**	1.011	0.894	0.803	0.726	0.658
**4**	1.019	0.902	0.810	0.734	0.666	**2**	1.013	0.896	0.804	0.728	0.660
**6**	1.023	0.905	0.814	0.737	0.668	**3**	1.014	0.897	0.805	0.729	0.661
**8**	1.027	0.910	0.817	0.740	0.671	**4**	1.017	0.899	0.808	0.731	0.663
**10**	1.032	0.914	0.822	0.744	0.675	**5**	1.020	0.905	0.814	0.736	0.668
**12**	1.038	0.919	0.826	0.748	0.679	**6**	1.033	0.918	0.827	0.749	0.685
**14**	1.045	0.926	0.833	0.754	0.684	**7**	1.042	0.927	0.835	0.758	0.690
**16**	1.050	0.930	0.836	0.758	0.688	**8**	1.047	0.931	0.839	0.762	0.693
**18**	1.054	0.934	0.840	0.762	0.692	**9**	1.050	0.935	0.843	0.766	0.697
**20**	1.057	0.937	0.844	0.765	0.696	**10**	1.053	0.937	0.845	0.768	0.699
**22**	1.061	0.941	0.847	0.768	0.699	**11**	1.055	0.939	0.848	0.770	0.701
**24**	1.065	0.945	0.850	0.771	0.701	**12**	1.057	0.941	0.850	0.772	0.704
**[Glycyl Dipeptide] = 0.010 mol·kg^{-1}**											
**2**	1.020	0.901	0.810	0.734	0.665	**1**	1.015	0.897	0.806	0.730	0.661
**4**	1.023	0.905	0.813	0.737	0.668	**2**	1.017	0.898	0.807	0.731	0.662
**6**	1.027	0.908	0.817	0.740	0.671	**3**	1.018	0.899	0.808	0.732	0.663
**8**	1.031	0.912	0.820	0.744	0.673	**4**	1.020	0.902	0.811	0.735	0.666
**10**	1.036	0.917	0.824	0.748	0.677	**5**	1.024	0.907	0.817	0.739	0.671
**12**	1.042	0.922	0.828	0.752	0.681	**6**	1.034	0.920	0.830	0.752	0.687
**14**	1.049	0.928	0.835	0.758	0.687	**7**	1.046	0.929	0.837	0.761	0.692
**16**	1.054	0.933	0.839	0.761	0.691	**8**	1.051	0.933	0.842	0.765	0.696

**18**	1.058	0.936	0.843	0.765	0.695	**9**	1.054	0.937	0.846	0.769	0.700
**20**	1.061	0.940	0.847	0.769	0.699	**10**	1.057	0.939	0.848	0.771	0.702
**22**	1.065	0.943	0.850	0.772	0.702	**11**	1.059	0.942	0.850	0.773	0.704
**24**	1.069	0.947	0.853	0.775	0.704	**12**	1.061	0.943	0.853	0.775	0.706
